# Opsoclonus-Myoclonus-Ataxia Syndrome (OMAS) Associated with SARS-CoV-2 Infection: Post-Infectious Neurological Complication with Benign Prognosis

**DOI:** 10.5334/tohm.580

**Published:** 2021-02-10

**Authors:** Enrique Urrea-Mendoza, Kimberly Okafor, Senthuran Ravindran, John Absher, Varun Chaubal, Fredy J. Revilla

**Affiliations:** 1 Prisma Health, Greenville, SC; 2 University of South Carolina School of Medicine-Greenville, Prisma Health, Greenville, SC

**Keywords:** Opsoclonus-Myoclonus Ataxia Syndrome, SARS-CoV-2, Movement Disorders, Post-infection

## Abstract

The novel coronavirus SARS-CoV-2 (severe acute respiratory syndrome coronavirus-2) is the cause of the COVID-19 pandemic [5]. SARS-Cov-2 demonstrates partial resemblance to SARS-CoV and MERS-CoV in phylogenetic analysis, clinical manifestations, and pathological findings [6, 7]. Reports emerging from China have described ataxia as a neurological symptom of the SARS-CoV-2 infection [5]. Opsoclonus consists of back-to-back multidirectional conjugate saccades without an inter-saccadic interval [8]. Myoclonus is defined as a sudden, brief, “shock-like”, nonepileptic involuntary movement [9], which has been described as a symptom of SARS-CoV-2 infection [10]. Opsoclonus-Myoclonus-Ataxia syndrome (OMAS) associated COVID-19 infection has been reported recently [11,12].

Coronavirus infection primarily affects the respiratory system, also spreading from the respiratory tract to the central nervous system (CNS) [1]. CNS manifestations include febrile seizures, convulsions, altered mental status, and encephalitis [2]. Although various papers have been published describing the neuroinvasive potential of SARS-CoV-2 [3,4], the capacity of SARS-CoV-2 to infect the CNS in humans is not well characterized, and further research regarding its unknown CNS pathology is required.

The novel coronavirus SARS-CoV-2 (severe acute respiratory syndrome coronavirus-2) is the cause of the COVID-19 pandemic [5]. SARS-Cov-2 demonstrates partial resemblance to SARS-CoV and MERS-CoV in phylogenetic analysis, clinical manifestations, and pathological findings [6,7]. Reports emerging from China have described ataxia as a neurological symptom of the SARS-CoV-2 infection [5]. Opsoclonus consists of back-to-back, multidirectional, conjugate saccades without an inter-saccadic interval [8]. Myoclonus is defined as a sudden, brief, “shock-like”, nonepileptic involuntary movement [9], which has been described as a symptom of SARS-CoV-2 infection [10].

Opsoclonus-Myoclonus-Ataxia syndrome (OMAS) associated COVID-19 infection has been reported recently [11,12].

A 32 year-old man presented with cough, fever, weakness, and loss of appetite. He denied changes in smell or taste. His father had similar symptoms 5 days earlier and both tested positive for SARS-CoV-2. He developed increasing fatigue and dyspnea but denied wheezing or chest tightness. He had watery, non-bloody diarrhea throughout the febrile period until his cough improved, and he became afebrile 11 days after diagnosis. On day 12 the patient developed ataxia and myoclonus. While hospitalized on days 17 to 20, evaluation revealed a chest x-ray consistent with viral pneumonia and a normal brain MRI. IgG antibodies were positive in serum. A lumbar puncture was not performed. Opsoclonus, myoclonus, and ataxia (***Videos 1*** and ***2***) caused the inability to ambulate without assistance. Treatment with clonazepam (1mg three times per day), divalproex (1,000 mg three times per day), and oral methylprednisolone (40 mg daily) were effective, allowing the patient to walk short distances without assistance. He had mild ataxia and minimal residual myoclonus at rest. Telehealth follow-up on day 24 demonstrated substantial improvement of gait and balance (***Video 3***). No opsoclonus was observed, and he demonstrated very mild ataxia and occasional myoclonus.

**Video 1:** Myoclonus and Ataxia.

**Video 2:** Opsoclonus.

**Video 3:** Day 24, substantial improvement of gait and balance. No opsoclonus was observed, very mild ataxia, and occasional myoclonus.

The etiology of Opsoclonus-Myoclonus-Ataxia syndrome includes paraneoplastic, parainfectious, toxic-metabolic, and idiopathic causes [8]. Infectious causes of this syndrome have been reported with herpes virus, arbovirus, and several parasitic infections [13,14,15]. This is a case of OMAS secondary to SARS-CoV-2 infection, and its clinical presentation suggests a post-infectious mechanism that is possibly antibody-mediated.

## References

[1] Asadi-Pooya AA, Simani L. Central nervous system manifestations of COVID-19: A systematic review. J Neurol Sci. 2020; 413: 116832 DOI: 10.1016/j.jns.2020.116832 32299017 PMC7151535

[2] Desforges M, Le Coupanec A, Dubeau P, et al. Human Coronaviruses and Other Respiratory Viruses: Underestimated Opportunistic Pathogens of the Central Nervous System? Viruses. 2019; 12(1). DOI: 10.3390/v12010014 PMC7020001 31861926

[3] Li YC, Bai WZ, Hashikawa T. The neuroinvasive potential of SARS-CoV2 may play a role in the respiratory failure of COVID-19 patients. J Med Virol. 2020; 92(6): 552–555. DOI: 10.1002/jmv.25728 32104915 PMC7228394

[4] Yachou Y, El Idrissi A, Belapasov V, Ait Benali S. Neuroinvasion, neurotropic, and neuroinflammatory events of SARS-CoV-2: understanding the neurological manifestations in COVID-19 patients. Neurol Sci. 2020 10; 41(10): 2657–2669. Epub 2020 Jul 28. PMID: 32725449; PMCID: PMC7385206. DOI: 10.1007/s10072-020-04575-3 32725449 PMC7385206

[5] Mao L, Jin H, Wang M, et al. Neurologic Manifestations of Hospitalized Patients With Coronavirus Disease 2019 in Wuhan, China. JAMA Neurol. Published online April 10, 2020. DOI: 10.1001/jamaneurol.2020.1127 PMC7149362 32275288

[6] Lu R, Zhao X, Li J, et al. Genomic characterisation and epidemiology of 2019 novel coronavirus: implications for virus origins and receptor binding. Lancet. 2020; 395(10224): 565–574. DOI: 10.1016/S0140-6736(20)30251-8 32007145 PMC7159086

[7] Xie M, Chen Q. Insight into 2019 novel coronavirus - An updated interim review and lessons from SARS-CoV and MERS-CoV [published online ahead of print, 2020 Apr 1]. Int J Infect Dis. 2020; 94: 119–124. DOI: 10.1016/j.ijid.2020.03.071 32247050 PMC7118633

[8] Oh SY, Kim JS, Dieterich M. Update on opsoclonus-myoclonus syndrome in adults. J Neurol. 2019; 266(6): 1541–1548. DOI: 10.1007/s00415-018-9138-7 30483882

[9] Burdick D, Agarwal P. 2016 Myoclonus. In Non–Parkinsonian Movement Disorders (eds D.A. Hall and B.R. Barton). DOI: 10.1002/9781118474075.ch4

[10] Rábano-Suárez P, Bermejo-Guerrero L, Méndez-Guerrero A, Parra-Serrano J, Toledo-Alfocea D, Sánchez-Tejerina D, Santos-Fernández T, Folgueira-López MD, Gutiérrez-Gutiérrez J, Ayuso-García B, González de la Aleja J, Benito-León J. Generalized myoclonus in COVID-19. Neurology. 2020 8 11; 95(6): e767–e772. Epub 2020 May 21. PMID: 32439821; PMCID: PMC7455360. DOI: 10.1212/WNL.0000000000009829 32439821 PMC7455360

[11] Sanguinetti S, Ramdhani RA. Opsoclonus Myoclonus Ataxia Syndrome Related to the Novel Coronavirus (COVID-19). J Neuroophthalmol. 2020 9 7 Epub ahead of print. PMID: 32925477. DOI: 10.1097/WNO.0000000000001129 PMC8366529 32925477

[12] Shah PB, Desai SD. Opsoclonus myoclonus ataxia syndrome (OMAS) in the setting of COVID-19 infection. Neurology. 2020 10 1:10.1212/WNL.0000000000010978. Epub ahead of print. PMID: 33004603. DOI: 10.1212/WNL.0000000000010978 33004603

[13] Chen Y, Chen D, Zhou X, Zhang H, Liao S, Xu Z, Xu P. Opsoclonus-myoclonus syndrome associated with herpes simplex virus infection: a case report. Int J Neurosci. 2020 3 9: 1–5. Epub ahead of print. PMID: 32116082. DOI: 10.1080/00207454.2020.1737531 32116082

[14] Afzal A, Ashraf S, Shamim S. Opsoclonus myoclonus syndrome: an unusual presentation for West Nile virus encephalitis. Proceedings (Baylor University. Medical Center). 2014; 27(2): 108–110. DOI: 10.1080/08998280.2014.11929073 24688189 PMC3954659

[15] Bose K, Saha S, Islam MR, Chakraborty C, Laskar M. Opsoclonus myoclonus ataxia syndrome due to falciparum malaria in two Indian children. Indian J Ophthalmol. 2016 11; 64(11): 852–854. PMID: 27958213; PMCID: PMC5200992. DOI: 10.4103/0301-4738.195611 27958213 PMC5200992

